# Incidental finding of a human-like tusavirus in a lamb with lip lesions and fatal pneumonia

**DOI:** 10.1099/jgv.0.001968

**Published:** 2024-03-05

**Authors:** Hannah Davies, Akbar Dastjerdi, David Everest, Tobias Floyd, Rachael Collins, Harriet McFadzean, Gábor Reuter, Rudolf Reichel

**Affiliations:** 1 Animal and Plant Health Agency (APHA)- Weybridge, Addlestone, Surrey, KT15 3NB, UK; 2 APHA-Starcross, Staplake mount, Starcross, Exeter, Devon, EX6 8PE, UK; 3 Department of Medical Microbiology and Immunology, Medical School, University of Pécs, Pécs, Hungary; 4 APHA-Thirsk, Wests House, Station Road, Thirsk North, Yorkshire, YO7 1PZ, UK

**Keywords:** tusavirus, pneumonia, lip lesions, protoparvovirus, pustular dermatitis, ovine parvovirus

## Abstract

Tusaviruses in the genus *Protoparvovirus* of family *Parvoviridae* were first identified in a diarrhoeic Tunisian child in 2014. Thereafter, high prevalence of a genetically similar virus was demonstrated in faeces from caprine and ovine species in Hungary. Here, we describe an investigation into the cause of scabby lip lesions in a 6 month-old lamb, submitted from a farm experiencing weight loss and scouring in lambs in England. Transmission electron microscopy visualised small circular particles of 18 and 22 nm in diameter in lip lesions identified as tusavirus and flumine parvovirus by Next Generation Sequencing. Liver, kidney, lung, small intestine content and faeces were also strongly positive for the tusavirus DNA as well as 10 % of faecal samples of the flock collected 2 months after the initial lip sampling. NS1 and VP1 amino acid sequences of this tusavirus displayed 99.5 and 92.89 % identity to those of a human tusavirus, respectively. These amino acid identities were at 95.5 and 89.68 % when compared to those of a goat tusavirus. Phylogenetic analysis of the NS1 and VP1 also grouped the virus in the genus *Protoparvovirus* and close to tusaviruses detected in human, ovine and caprine species. Wider surveillance of the virus indicated a broader geographical distribution for the virus in England. Histology of the lip tissue revealed localised areas of epidermal hyperplasia and hyperkeratosis affecting haired skin, with mild leucocyte infiltration of the subjacent dermis, but no changes to implicate virus involvement. Flumine parvovirus was concluded to be an environment contaminant. Broader studies in prevalence of these virus in UK sheep flocks and human population, animal models and experimental infections could provide insights into the pathogenesis of these novel viruses and their zoonotic potential.

## Introduction

Human stool-associated tusaviruses belong to the genus *Protoparvovirus*, subfamily *Parvovirinae* and family *Parvoviridae;* identified first in a diarrhoeic child from Tunisia in 2014 [1]. They are small non-enveloped viruses containing a single-stranded DNA genome of 4–6 kb. Studies limited to this Tunisian case and two additional cases, Finnish adults in their twenties with gastroenteritis [2], suggest that tusavirus infection may be associated with gastrointestinal disease in humans. In recent years, limited serological studies have also been carried out to assess prevalence of human protoparvoviruses and found evidence of tusavirus IgG. One study from 2016 found only one tusavirus antibody positive sample, out of 228 children tested, and no seropositive was identified in the 180 adults screened [3]. A further study found no evidence of tusavirus IgG in individuals from Finland, Iran, Iraq, Kenya, and the USA [4]. This negligeable level of seropositivity has led to the notion that the virus may have originated from an animal source and are detected in human stools due to their resilient capsid structure.

Previous serological studies in the United Kingdom indicate high prevalence of ovine parvoviruses in British and Scottish sheep flocks, however, the genetic nature of this parvovirus remains unknown [5,6]. A recent study from Hungary, demonstrated high prevalence of a virus genetically similar to the human stool-associated tusavirus in faeces from caprine and ovine species. However, no seroprevalence of the viruses in humans was carried out in the study to correlate their prevalence to those of the animals. In the study, positive detections were made in both healthy and diarrhoeic animals, so no causative link to the disease could be established [7].

In animals, protoparvoviruses such as porcine parvovirus (ungulate protoparvovirus 1), feline parvovirus (also known as feline panleukopenia virus) and canine parvovirus can cause severe disease and mortality resulting in major economic loss and welfare issues in pigs, dogs and cats respectively [8,10]. Additionally, canine parvovirus has been shown to cause erythematous cutaneous lesions in dogs [8,11, 12]. The significance of tusavirus infections in humans and animals remains undetermined, therefore, it is crucial to gain further insight into the biology of this novel parvovirus.

This paper describes the detection of tusavirus in a 6 month-old lamb with lip lesions submitted to investigate the causes of diarrhoea, poor growth and mortality in a group of lambs. We present the full genome sequence of this tusavirus detected in the lip lesions and other organs, its phylogenetic analysis and prevalence within the affected farm. Moreover, we provide the first preliminary evidence of the virus prevalence in the wider domestic sheep population in England.

## Methods

The submitted lamb carcase was identified, weighed, necropsied with gross changes described, and samples collected for further testing as per the APHA protocol for diagnostic carcase submissions. The post-mortem examination followed the standard protocol used in APHA VICs, ensuring a systematic approach and comprehensive assessment of all body systems.

### Next generation sequencing (NGS) and phylogenetic analysis

Lip lesions, removed at post-mortem using a disposable scalpel, were homogenised in 300 µl PBS using the GentleMACS dissociator, centrifuged at 5000 ***g*** for 3 min and then nucleic acid was extracted from 140 µl of supernatant using QIAamp Viral RNA kit (Qiagen) following the kit instructions. Nucleic acid was submitted for sequencing on Illumina NextSeq at the Central Sequencing Unit, APHA-Weybridge. Raw sequencing data was assembled against all virus reference sequences from GenBank using NGen17.5 (DNASTAR) metagenomics pipeline. Phylogenetic analysis was completed on the protein sequence of coding sequence (CDS) of NS1 and VP1 and partial amino acid sequences of VP1. The sequences were aligned using MAFFT and a model test was completed in mega 11 to identify the evolutionary model that best fits the data. Phylogenetic trees were constructed in mega 11 software.

### Nucleic acid extraction and polymerase chain reaction (PCR)

Nucleic acid extractions from lamb tissues were done as outlined above for lip lesions. Faecal samples were extracted using EZ1 mini kit (Qiagen) following universal tissue, total RNA protocol on the EZ1 Advanced XL instrument (Qiagen). Nucleic acid was tested for tusavirus DNA using a qPCR with primers and probe targeting a conserved region of the viral VP1 gene. The primers and probe sequences for the qPCR were as follows: forward primer 5′-ATCTTCAGGAGGAGGTGCT-3′, reverse primer 5′-TAGTCTAGAGAGTCTGGTCT-3′, probe [6FAM]-GTGTCTACTGGTGATTTTGACAATACT-[BHQ1]. The qPCR was performed using QuantiFast Pathogen PCR kit (Qiagen) and the mastermix was consisted of 0.5 µl of each 20 µM primers, 0.05 µl of 100 µM probe, 5 µl 5×QuantiFast pathogen mix, 16.95 µl molecular biology grade water and 2 µl of nucleic acid. The thermocycling conditions for the qPCR were 95 °C for 5 min followed by 42 cycles of 95 °C for 15 s and 60 °C for 30 s with fluorescence being captured at the end of each cycle. The 18S qPCR primers and probe were as described by Lew *et al*. [13] and the qPCR was conducted as above. The ΔCt was calculated by subtracting 18S Ct value from that of the tusavirus Ct value for each tusavirus positive sample. The additional ovine tusavirus sequence (strain 0107, accession number OR734234) was obtained using primers and PCR conditions as described by Reuter *et al*. [7] followed by in-house Sanger sequencing. Poxvirus PCR was done according to Li *et al*. [14].

### Other investigations

#### Parasitology

Estimated total worm count was undertaken on faeces as described [15].

#### Electron microscopy

Sample extractions were undertaken on unfixed lip skin samples in a Class 1 MSC. Examinations were by negative contrast stain transmission electron microscopy (TEM) using 3 mm diameter Copper/Rhodium (100μ mesh) support grids for the analyses. Each grid was pre-treated by immersion into 0.4 % (w/v) formvar in chloroform, followed by carbon coating, to provide a stable sample platform. Grid treatment was completed, by plasma glow discharge, to ensure hydrophilic grids. Each sample (approx. 0.50 g) was ground in 2 cm^3^ of 0.1M Sorenson’s phosphate buffer (pH 6.6), to form a suspension. An aliquot of each sample suspension (50 µl) was pipetted onto dental wax and a support grid placed copper side upwards onto each aliquot for approximately 30 s and excess sample removed by wicking dry. Each grid was placed as before onto a drop of 2 % phosphotungstic acid (pH 6.6) for approximately 10 s to counterstain the grid and excess stain removed by wicking dry. Sample analysis was performed on a TECNAI Bio-Twin 12 TEM (Thermo fisher, FEI Company, USA), typically at ×6800 magnification at 80 kv. Confirmation of viral particle presence was by size, shape and available surface morphology and digital images were captured using an AMTXR60 camera (Deben, UK). Analysis time was standardised as 20 min viewing of each sample grid or checking 25 grid squares whichever was shortest, to observe virus particle presence.

#### Histology

Formalin-fixed tissues were processed by a routine method and sections were stained with haematoxylin and eosin for microscopic examination.

#### Bacteriology

Aerobic bacterial cultures were prepared on lung samples by routine methods using sheep blood agar and MacConkey’s agar plates and incubated at 37 °C for 24 h. Mycoplasma culture and PCR was undertaken according to APHA protocols.

#### Surveillance samples and DNA sequencing

Prevalence of the virus on this farm was assessed through collection and testing of faecal samples. In total 40 faecal samples were submitted, ten samples collected 2 months after the initial case with a further ten from young lambs and ten from adults in the following months. A final ten samples were also collected 10 months later. To assess the wider virus prevalence in sheep flocks in England, samples from routine post-mortem cases submitted to APHA VICs were checked by the qPCR developed in this project. From each post-mortem, the following tissues were submitted: liver, lung, kidney, small intestinal content, spleen, and tonsil. Partial VP1 sequencing of subsequent positive cases was achieved using PCR described by Reuter *et al*. [7] and sequenced via Sanger sequencing at APHA-Weybridge. The amino acid sequences were aligned using Clustal W in MegAlign Pro v.17 (DNASTAR) and phylogenetic tree constructed using mega 11 software.

## Results

### Clinical history

The flock had experienced diarrhoea and loss of body condition affecting 20 of 60 lambs, with two lambs dying. An on-farm post-mortem examination of the first lamb to die revealed pneumonia. The second lamb to die was that submitted. Crusting lesions around the lips were also noted in a proportion of lambs in the flock, but for many of the lambs, these lesions had resolved by the time of investigation. Worm egg count performed by another laboratory on a pooled faecal sample had demonstrated a heavy worm burden, and so the flock had been treated with monepantel 5 days prior to submission, and with moxidectin twice in the previous 2 months. All lambs were home bred, but some of the ewes (dams) were purchased. The lambs were being sold as finished lambs, but about 150 lambs remained on farm (across all management groups) through investigation. The lambs were grazing permanent pasture with no significant amount of thistle or other prickly plants in the pasture. No on-farm vaccinations were employed.

### Gross pathology and histology

The carcase of an ewe lamb which was found dead was submitted to APHA Starcross for diagnostic investigation. This was the second lamb in the group to die. The body condition of the lamb was poor, with scant fat reserves, and there was significant lung pathology, characterised by consolidation and dark red discolouration of the cranial and middle lung lobes, oedema of the caudal lung lobes and fibrin deposition on the pleura. There were also crusting lesions on the lips – reminiscent of orf parapoxvirus infection – and the contents of the large intestine were liquid. There were no significant gross pathological findings in the musculoskeletal, cardiovascular, lymphoreticular, endocrine, urinary or reproductive systems.

Histopathological investigation was limited to the alimentary tract: including samples of lip, abomasum and intestine. Microscopic examination of the crusting lesions on the lip margin revealed epidermal hyperplasia and orthokeratosis, with mild leucocyte infiltration of the subjacent dermis. Septated bacterial filaments, forming tracks of diplococci morphologically consistent with *Dermatophilus congolensis*, were embedded in the surface crusts. Examination of sections of abomasum, small intestine and large intestine revealed a mild eosinophilic, lymphocytic and plasmacytic abomasitis and enterocolitis, with hyperplasia and mucous metaplasia of the abomasal mucosa. Low numbers of *Eimeria* spp. coccidian parasites were present in the mucosa of the small intestine.

### Non-viral laboratory findings

Parasitological examination on faeces collected from the fleece of this animal detected 50 Trichostrongyle-type eggs per gram, 250 Strongyle-type eggs per gram and 3400 coccidian oocysts per gram of faeces. *Mannheimia haemolytica* was isolated in mixed flora from swabs of the lung. *Mycoplasma ovipneumoniae* and *Mycoplasma arginine* were also detected in lung swabs by PCR.

### TEM examination

**Fig. 1.**
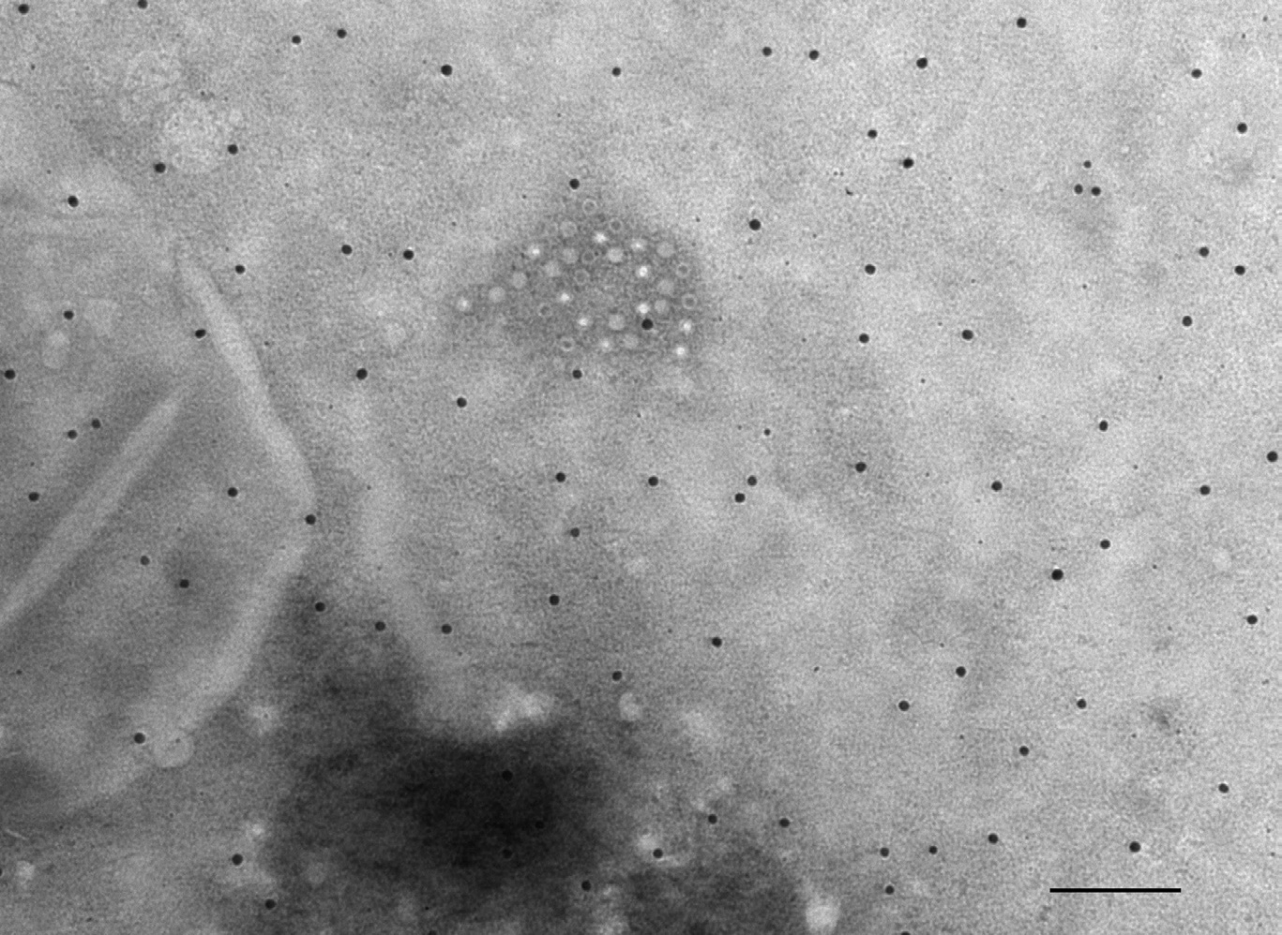
Electron micrograph of tusavirus-like particles from scabby lip lesions of a 6 month-old lamb visualized by TEM. Mag. bar=100 nm.

Examination of the lip lesions at 80kv revealed many groups of small circular particles detected throughout the sample grid. Over 40 digital images were taken of these particle groups, representing a sustained presence throughout the sample grid. The particles were predominantly 18 to 22 nm in diameter, some being white solid particles, while others presented as a capsid form with a dark centre. Their size, shape and available morphology were consistent with that of a parvovirus (Fig. 1). No parapoxvirus particle presence was detected, neither was parapoxvirus DNA (including Orf virus) by PCR as initially suspected. Faecal material was negative for a virus particle possibly due to detection limit of TEM or other unknown reasons.

### NGS data and phylogenetic analysis

A total of 633 589 out of 22 311 008 sequence reads mapped to a tusavirus genome with median coverage of 30 339.6. The 4751-nucleotide genome of strain 0215 (accession number OR734234) contained a 3′ untranslated region (419 nt, the longest known sequence to date), complete non-structural protein 1 (NS1) replicase (625 amino acid [aa]), viral protein 1 (VP1, 715 aa) and VP2 (564 aa) genes, and a 5′ untranslated region (185 nt). This tusavirus has a potential upstream start codon MSS in a weaker Kozak consensus sequence than MAQ in NS1, which we selected as the start codon. All of the characteristic aa sequence motifs present in stool-associated human tusavirus were also identifiable in strain 0215, including the same start codons in NS1 and VP1. Analysis of the aa sequence of NS1 and VP1 CDS of this strain revealed 99.5 and 92.89 % identity to those of a stool-associated human tusavirus (accession number KJ495710) respectively. These amino acid identities were at 95.5 and 89.68 % when compared to those of a goat tusavirus (accession number OL692339). Analysis of partial NS1 sequence, to allow for inclusion of published sheep tusavirus sequences, found equal amino acid identity (97.6 %) to the published sheep tusavirus (accession numbers OL692343-OL692347) and the stool-associated human tusavirus. Sequencing of partial VP1 from surveillance case 0107 (accession number OR734235) also identified a tusavirus with 97.6 % amino acid identity to the 0215 case. Comparing the amino acid sequences of the VPs of the stool-associated human tusavirus (NC_075988), goat tusavirus (OL692339) and the study strain 0215 (OR734234), the changes in amino acid motifs are grouped at variable regions (VR) corresponding to the surface-exposed immunogenic loops of tusaviruses [16] especially in VR1, VR3-VR9 and HI VR loop.

**Fig. 2.**
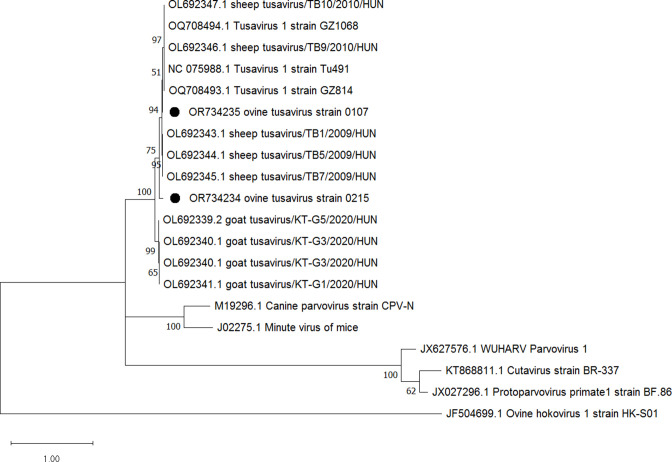
Phylogenetic analysis of partial amino acid sequence of VP1 of protoparvovirus detected in this study and those reported previously. The sequence obtained in this study is indicated by the black circle. The maximum-likelihood tree was constructed in mega11 using LG with freqs (+F), gamma distribution (+G) and invariant sites (+I) model. Bootstrap values of >60 % are indicated at the nodes.

Phylogenetic analysis of the NS1 and VP1 CDS (supplementary data, available in the online version of this article) and partial NS1 sequences (Fig. 2) accordingly grouped the two viruses in the genus *Protoparvovirus* and close to tusaviruses detected in human, ovine and caprine species. NGS data analysis also revealed a second parvovirus-like virus identified as flumine parvovirus (accession number OR751333) with 240 of the sequence reads were mapped to this virus. The sequences were covering at least half of the virus genome of the published flumine parvovirus accession number OM954006.1, albeit with a low median coverage of 9.59. No other viruses’ genetic materials could be identified in the raw sequence data.

### Ovine tusavirus prevalence

In total, five tissue samples (lip, liver, kidney, lung, and spleen) and faeces from this 6 month-old lamb were tested via qPCR for tusavirus DNA and all samples were strongly positive for the virus DNA with Ct values ranging from 14 to 22 (ΔCt of −5.79 to 9.27) (Tables1  2). As part of assessing the prevalence within the affected farm, 40 faecal samples were also collected in the months following identification of this tusavirus. Of the 40 faeces samples, five tested positive for tusavirus DNA, three of the ten samples taken 2 months with the remaining two taken 10 months after initial lip sampling (Table 2). Of the nine tissue submissions received as part of a wider surveillance between April and July 2023, three submissions had tissues which were positive for tusavirus DNA and were not confined to a single geographical location (Tables1  2).

**Table 1.** Overview of cases investigated for ovine tusavirus presence using a real time qPCR developed in this study. In total, samples from nine farms located in different geographical regions of England were included in this study. The qPCR Ct values were normalised to those of 18S for comparative analysis

Case ID	Sample type	Sampling date	Region	No. of samples	No. of positives	Ranges of normalised ct values (∆Ct)§
0215*	Lip and PM tissues†	November, 2022	Devon	6	6	−3.03 to 9.27
0262*	Faeces	January, 2023	Devon	10	3	3.99 to 4.95
0067*	Faeces	April, 2023	Devon	10	0	n/a
0068*	Faeces	April, 2023	Devon	10	0	n/a
0049	PM tissues‡	April, 2023	York	6	0	n/a
0055	PM tissues	April, 2023	Suffolk	6	0	n/a
0067	PM tissues	April, 2023	Cambridge	6	0	n/a
0107	PM tissues	April, 2023	Harrogate	6	6	8.08 to 23.84
0078	PM tissues	May, 2023	York	17¶	0	n/a
0066	PM tissues	May, 2023	York	6	3	17.42 to 25.41
0077	PM tissues	May, 2023	York	6	1	25.08
0006	PM tissues	May, 2023	York	6	0	n/a
0057*	Faeces	September, 2023	Devon	10	2	4.7 to 7.24

*Original farm.

†Lip, liver, kidney, lung, small intestinal content, and faeces.

‡Post mortem (PM) tissues were liver, lung, kidney, spleen, tonsil, and small intestinal content.

§Ct values for ovine tusavirus were normalised to those of 18S.

¶PM tissues from three animals, tonsil was missing from one set of tissues.

n/a; not applicable.

**Table 2.** Details of ovine tusavirus positive samples identified in this study. An ovine tusavirus and an 18S real time qPCRs were used to test the samples and ovine tusavirus Ct values were normalised to those of 18S (ΔCt) for each sample. Geographical location of sheep flocks is also given in the table

Samples	Initial case	Wider surveillance cases		
	0215-DevonCt (∆Ct)	0107-HarrogateCt (∆Ct)	0066-YorkCt (∆Ct)	0077-YorkCt (∆Ct)
Lip	14 (-3.03)*	n/a	n/a	n/a
Liver	17 (1.39)	37.3 (20.07)	34.48 (19.54)	No Ct
Kidney	20 (3.87)	33.86 (18.63)	31.62 (17.42)	39.4 (25.08)
Lung	21.5 (4.93)	41.4 (23.84)	No Ct	No Ct
Tonsil	n/a	36.9 (21.19)	No Ct	No Ct
Spleen	n/a	28.32 (16.49)*	38.2 (25.41)	No Ct
Small Intestine	22 (9.27)	38 (23.84)	No Ct	No Ct
Faeces	19 (-5.79)	n/a	n/a	n/a

* ; Samples from which the sequence data were derived.

n/a; no sample was available.

## Discussion

Here we report for the first time, the detection of a tusavirus closely related to stool-associated human tusavirus and a flumine parvovirus from ovine species in the UK. Considering the number of sequences reads mapped to each virus, along with conditions on the farm, we concluded the likelihood of the flumine parvovirus to be a water contaminant and not causing infection in the lamb. Flumine parvoviruses have been found in river waters [17] and their inclusion into the *Parvoviridae* family has not yet been approved by ICTV. These lambs were at pasture and their only water source was a stream that ran through the farm, and which had a human sewage works beside it. Sheep drank downstream from the treated sewage discharge point. Further reports detailed overspills of untreated sewage and the weather was wet at times in November 2022. No viral surveillance of stream water, to our knowledge, had been carried out by the provider of the utilities. The identified ovine tusavirus exhibited the highest amino acid sequence identity to those of stool-associated human tusavirus for the NS1 and VP1 genes and to other goat and sheep sequences reported using partial VP1 sequence. Whilst the origin of stool-associated human tusavirus remains unknown, it is believed to be a zoonotic pathogen [7] that may have originated from animal species. So far, no serological data is available to indicate prevalence of the viruses in the UK human population. However, if the viruses indeed originated from animal species, i.e. small ruminants, their prevalence should be relative to those of sheep flocks among abattoir workers, veterinarians and sheep farmers. Further seroprevalence and genomic studies in both human and small ruminants would shed insights into the epidemiology of these potentially zoonotic viruses.

The cause of the lamb’s death in this case was determined to be pneumonia due to infection with *Mannheimia haemolytica* (Pasteurellosis). Infection with *Mycoplasma ovipneumoniae* may well have contributed to the development of pneumonia. Endoparasitism had already been diagnosed in the flock and was the most likely explanation for the poor condition and diarrhoea observed in the submitted lamb. The low faecal worm egg count can be attributed to recent anthelmintic treatment. The coccidian infection was not deemed to be clinically significant. Histopathology of the lip lesions revealed inflammation and infection of the skin surface by bacteria morphologically typical of *Dermatophilus congolensis*. Dermatophilosis typically follows chronic wetting of the skin and fleece and is more common in young or immunosuppressed individuals [18]. Damage to the epidermal barrier may precipitate the infection, including ectoparasite damage, mechanical trauma from grazing stubble or damage caused by epitheliotropic viruses like orf parapoxvirus. However, there were no histological changes in the sections of skin examined to implicate virus involvement. Furthermore, examination of the suspected lip lesion by TEM, NGS and PCR effectively ruled out orf virus infection. The possibility of mild initiating or underlying lesions caused by the tusavirus can’t be ruled out by this investigation and examination of further cases is required. Histological examination of tissues from the remainder of the gastrointestinal tract did not reveal any lesions consistent with pathogenic virus infection – but evaluation for more subtle lesions was compromised by the suboptimal preservation of the enteric tissues.

The pathogenic potential of human parvovirus B19 has long been established [19,20]. Other recently discovered protoparvoviruses have been linked to systemic infection and skin lesions in humans [20,21]. Bufavirus (BuV) was originally discovered in humans [22] and BuV-like viruses have been detected in blood and spleen of rhesus monkeys and baboons [23,24]. Cutavirus (CuV) was discovered in 2016 [25] and virus DNA has been detected both in the diarrhoeal samples of children and in skin samples of patients with cutaneous T-cell lymphoma and melanoma. However, a more recent study found no evidence for the role of cutavirus in malignant melanoma [26]. No CuV has yet been reported from animal species.

Screening of the original farm detected tusavirus DNA 2 months after the initial case and then following a resurgence of diarrhoea ten-months later demonstrating continued circulation of the virus in the flock and/or reintroduction from the sewage works. Surveillance for tusavirus infection in the wider sheep population identified the presence of the virus DNA in geographically distant regions of England and circulation of at least another strain of the virus. Majority of the tusavirus positive tissue samples were borderline positive and unsuitable for sequencing to ascertain their diversity in England. The infectivity of the viruses in the faeces for both ovine and non-ovine species also has yet to be determined. It is notable to highlight that this is the first report of tusavirus DNA detection in tissues implying a systemic infection, previous findings of the virus DNA have only been from faeces. Further work is required to improve our understanding of tusavirus biology and whether it has potential to produce disease in sheep or is an ovine adapted virus. Further studies could also investigate the serological prevalence of the virus in UK sheep flocks and human population, virus isolation and experimental infection of animals to gain insights into pathogenesis of the virus.

## supplementary material

10.1099/jgv.0.001968 Uncited Supplementary Material 1.

## Abbreviations

BuV: bufavirus
CDS: coding domain sequence
Ct: cycle threshold
CuV: cutavirus
MSC: microbiological safety cabinet
NGS: next generation sequencing
NS: non-structural viral protein
ORF: open reading frame
PCR: polymerase chain reaction
TEM: transmission electron microscopy
VP: structural viral protein
VR: variable region
