# Supplementary material for: Incidental finding of a human-like tusavirus in a lamb with lip lesions and fatal pneumonia
Source: J Gen Virol. 2024 Mar 5;105(3):001968. doi: 10.1099/jgv.0.001968 (PMC10999738; doi:10.1099/jgv.0.001968)
Supplement: Uncited Supplementary Material 1. [file jgv-105-01968-s001.pdf]

Supplementary data:

Phylogenetic analysis of complete amino acid sequences of NS1 (A) and VP1 (B). The maximum-likelihood tree was constructed in MEGA11 using LG with freqs (+F), gamma distribution (+G) and invariant sites (+I) model. Bootstrap values of >60% are indicated at the nodes. The sequence obtained in this study is indicated by the black circle.

A:

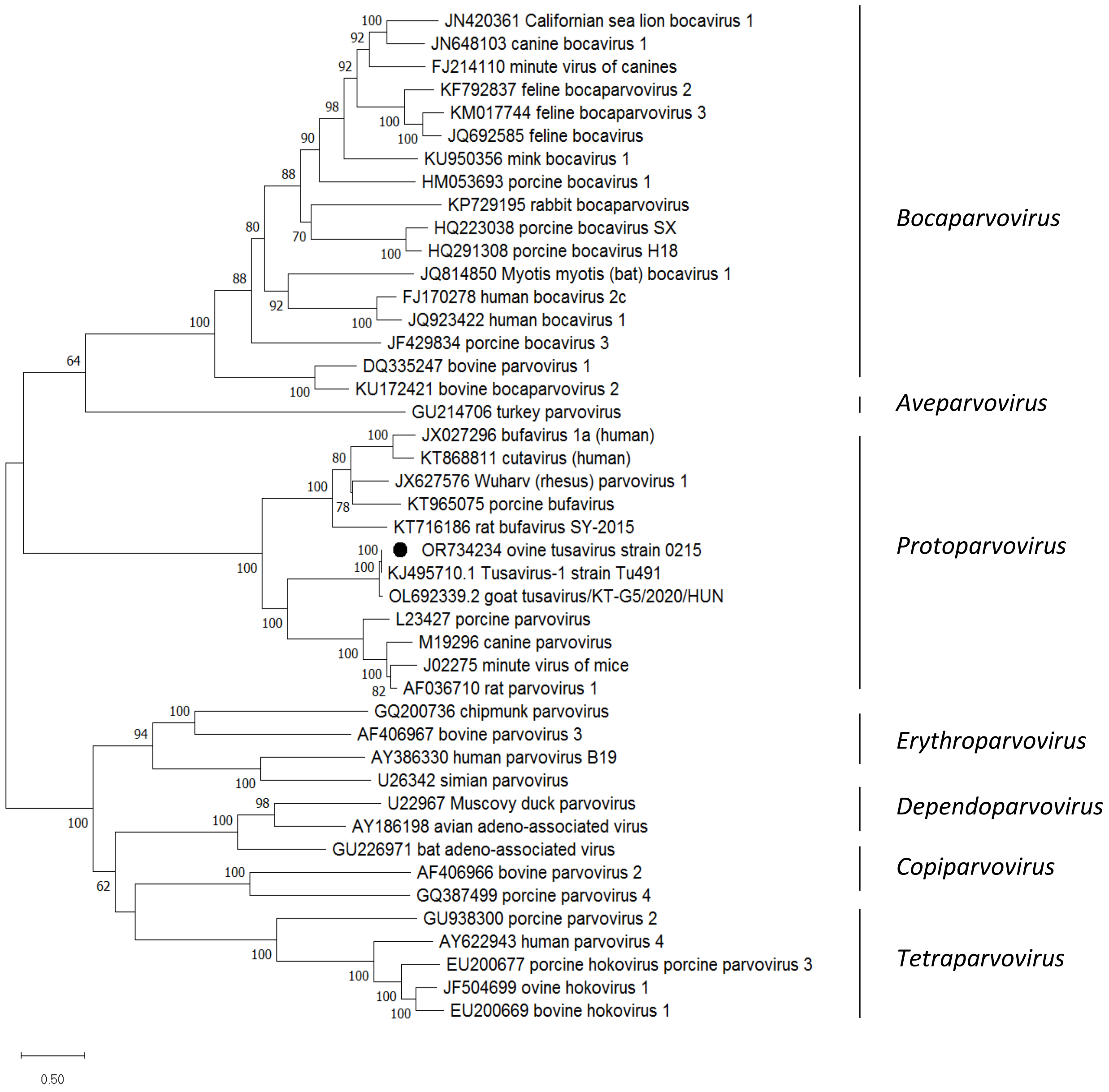

B:

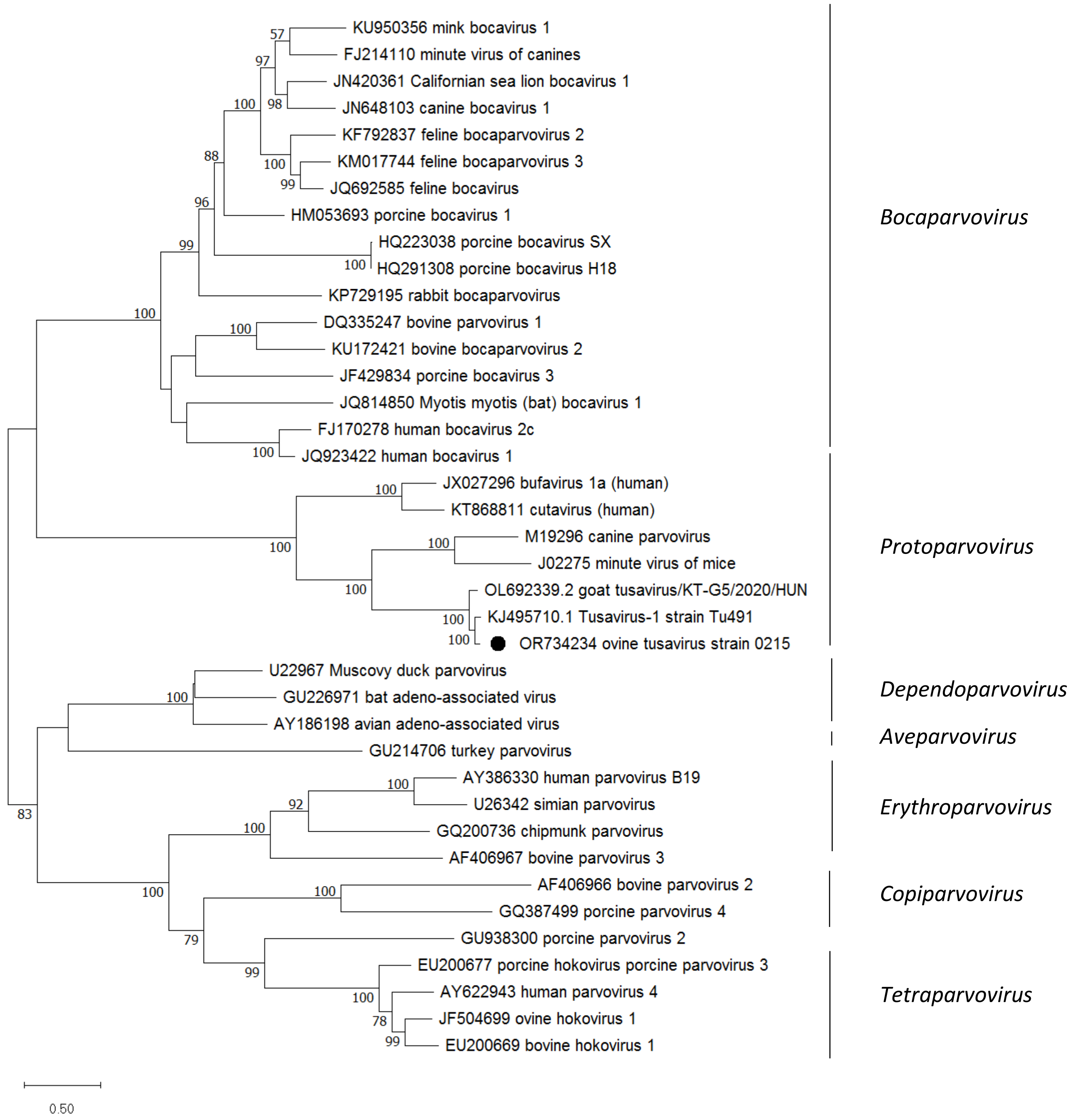

KU950356 mink bocavirus 1
FJ214110 minute virus of canines
JN420361 Californian sea lion bocavirus 1
JN648103 canine bocavirus 1
KF792837 feline bocaparvovirus 2
KM017744 feline bocaparvovirus 3
JQ692585 feline bocavirus
HM053693 porcine bocavirus 1
HQ223038 porcine bocavirus SX
HQ291308 porcine bocavirus H18
KP729195 rabbit bocaparvovirus
DQ335247 bovine parvovirus 1
KU172421 bovine bocaparvovirus 2
JF429834 porcine bocavirus 3
JQ814850 Myotis myotis (bat) bocavirus 1
FJ170278 human bocavirus 2c
JQ923422 human bocavirus 1
JX027296 bufavirus 1a (human)
KT868811 cutavirus (human)
M19296 canine parvovirus
J02275 minute virus of mice
OL692339.2 goat tusavirus/KT-G5/2020/HUN
KJ495710.1 Tusavirus-1 strain Tu491
OR734234 ovine tusavirus strain 0215
U22967 Muscovy duck parvovirus
GU226971 bat adeno-associated virus
AY186198 avian adeno-associated virus
GU214706 turkey parvovirus
AY386330 human parvovirus B19
U26342 simian parvovirus
GQ200736 chipmunk parvovirus
AF406967 bovine parvovirus 3
AF406966 bovine parvovirus 2
GQ387499 porcine parvovirus 4
GU938300 porcine parvovirus 2
EU200677 porcine hokovirus porcine parvovirus 3
AY622943 human parvovirus 4
JF504699 ovine hokovirus 1
EU200669 bovine hokovirus 1
Bocaparvovirus
Protoparvovirus
Dependoparvovirus
Aveparvovirus
Erythroparvovirus
Copiparvovirus
Tetraparvovirus
0.50
